# Radiomic Assessment of Radiation-Induced Alterations of Skeletal Muscle Composition in Head and Neck Squamous Cell Carcinoma within the Currently Clinically Defined Optimal Time Window for Salvage Surgery—A Pilot Study

**DOI:** 10.3390/cancers15184650

**Published:** 2023-09-20

**Authors:** Matthias Santer, Herbert Riechelmann, Benedikt Hofauer, Joachim Schmutzhard, Wolfgang Freysinger, Annette Runge, Timo Maria Gottfried, Philipp Zelger, Gerlig Widmann, Hanna Kranebitter, Stephanie Mangesius, Julian Mangesius, Florian Kocher, Daniel Dejaco

**Affiliations:** 1 Department of Otorhinolaryngology-Head and Neck Surgery, Medical University of Innsbruck, 6020 Innsbruck, Austria; matthias.santer@i-med.ac.at (M.S.); herbert.riechelmann@i-med.ac.at (H.R.); benedikt-gabriel.hofauer@i-med.ac.at (B.H.); joachim.schmutzhard@i-med.ac.at (J.S.); wolfgang.freysinger@i-med.ac.at (W.F.); annette.runge@tirol-kliniken.at (A.R.); timo.gottfried@i-med.ac.at (T.M.G.); 2 Department for Hearing, Voice and Speech Disorders, Medical University of Innsbruck, 6020 Innsbruck, Austria; philipp.zelger@i-med.ac.at; 3 Department of Radiology, Medical University of Innsbruck, 6020 Innsbruck, Austria; gerlig.widmann@i-med.ac.at (G.W.); hanna.kranebitter@student.ac.at (H.K.); 4 Department of Neuroradiology, Medical University of Innsbruck, 6020 Innsbruck, Austria; stephanie.mangesius@i-med.ac.at; 5 Department of Radiation-Oncology, Medical University of Innsbruck, 6020 Innsbruck, Austria; julian.mangesius@i-med.ac.at; 6 Department of Internal Medicine V (Hematology and Oncology), Comprehensive Cancer Center Innsbruck (CCCI), Medical University of Innsbruck, 6020 Innsbruck, Austria

**Keywords:** head and neck neoplasms, head and neck cancer, head and neck squamous cell carcinoma, radiotherapy, radiochemotherapy, salvage surgery, time interval, body composition, skeletal muscle, computed tomography scan, radiomics

## Abstract

**Simple Summary:**

Radiochemotherapy (RCT) in patients with locally advanced head and neck squamous cell carcinoma (HNSCC) causes side effects in healthy tissue such as the sternocleidomastoid muscle (SCM). These side effects depend on the interval between completion of RCT and restaging CT. For salvage surgery, the optimal time window for surgery is clinically postulated at between 6 and 12 weeks after completion of RCT. Thus, no extensive tissue fibrosis is to be expected. This interval is based on clinical studies exploring surgical complications. Studies directly exploring radiation-induced changes of the SCM in HNSCC patients are sparse. This study applied radiomics to quantify radiation-induced changes in the SCM and paravertebral musculature (PVM). In 98 locally advanced HNSCC patients, three radiomic key features (volume, mean positivity of pixels, uniformity) were analyzed in CT scans before and in the mean 8 weeks after treatment. No significant changes in radiomic key features were observed after adjustment for changes in body mass index (BMI). These data support the clinically postulated time window for salvage surgery of 6 to 12 weeks.

**Abstract:**

Patients with locally advanced head and neck squamous cell carcinoma (HNSCC) frequently require primary radiochemotherapy (RCT). Despite intensity modulation, the desired radiation-induced effects observed in HNSCC may also be observed as side effects in healthy tissue, e.g., the sternocleidomastoid muscle (SCM). These side effects (e.g., tissue fibrosis) depend on the interval between the completion of RCT and restaging CT. For salvage surgery, the optimal time window for surgery is currently clinically postulated at between 6 and 12 weeks after completion of RCT. Thus, no extensive tissue fibrosis is to be expected. This interval is based on clinical studies exploring surgical complications. Studies directly exploring radiation-induced changes of the SCM in HNSCC patients are sparse. The present study quantified tissue alterations in the SCM and paravertebral musculature (PVM) after RCT, applying radiomics to determine the optimal time window for salvage surgery. Three radiomic key parameters, (1) volume, (2) mean positivity of pixels (MPP), and (3) uniformity, were extracted with mint Lesion^TM^ in the staging CTs and restaging CTs of 98 HNSCC patients. Of these, 25 were female, the mean age was 62 (±9.6) years, and 80.9% were UICC Stage IV. The mean restaging interval was 55 (±28; range 29–229) days. Only the mean volume significantly decreased after RCT, from 9.0 to 8.4 and 96.5 to 91.9 mL for the SCM and PVM, respectively (both *p* = 0.007, both Cohen’s d = 0.28). In addition, the mean body mass index (BMI) decreased from 23.9 (±4.2) to 21.0 (±3.6) kg/m² (*p* < 0.001; Cohen’s d = 0.9). The mean BMI decreased significantly and was correlated with the volume decrease for the SCM (r = 0.27; *p* = 0.007) and PVM (r = 0.41; *p* < 0.001). If *t*-test *p*-values were adjusted for the BMI decrease, no significant change in volumes for the SCM and PVM was observed (both *p* > 0.05). The present data support the clinically postulated optimal interval for salvage surgery of 6 to 12 weeks.

## 1. Introduction

Most patients with locally advanced head and neck squamous cell carcinoma (HNSCC) require multimodality treatment [1,2], frequently consisting of surgery followed by radiotherapy (RT) or primary concurrent radiochemotherapy (RCT) [3]. While the RT part directly targets primary tumors and involved cervical lymph nodes, the chemotherapy part aims at increasing radiosensitivity and targets circulating tumor cells [2,3,4].

Modern photon-based radiation aims at primarily damaging primary tumors and suspicious cervical lymph nodes by more focused mechanisms almost exclusively at the site of radiation [5,6]. Despite the introduction of three-dimensional and conformal intensity-modulated radiation modalities, radiation-induced changes remain a challenge in RCT of HNSCC patients [7]. Thus, direct and desired effects of radiation observed in cancer cells [6] may also be observed as undesired effects in otherwise healthy tissue, which lies adjacent to but is not infiltrated by HNSCC (e.g., salivary glands, mucosal membranes, or skeletal muscle) [5]. For skeletal muscle, these alterations are mediated via tissue or stem cell injury, cellular signal pathway alterations, and (epi)genetic changes [8,9,10,11,12,13]. In contrast, the chemotherapy of a primary RCT was not previously observed to induce similar and significant tissue alterations [3,4].

The occurrence of these tissue alterations was previously found to depend on the interval between the end of RCT and the time of tissue assessment [14,15]. During the first weeks after RCT, muscular inflammation was found to lead to interstitial edema [16]. In contrast, in the months after RCT, a shift towards muscular fibrosis [14,15] due to misdirected wound healing [17,18] can be observed. Thus, ultimately, functional impairment may be observed [19,20,21].

Despite these functional impairments [19,20,21], this temporal dependence of tissue alterations after primary RCT also becomes relevant in the context of salvage surgery [22,23]. Reports on the incidence of persistent primary tumors or cervical lymph nodes after primary RCT range from 22 up to 40% [24,25,26]. Irrespective of the risk–benefit ratio of salvage surgery after primary RCT (i.e., missing microscopic residual disease if not performed vs. probability of overtreatment if performed) with possible surgical complications, the optimal time window for salvage surgery is currently clinically defined as an interval between 6 and 12 weeks after the completion of primary RCT [27]. For this specific time window, no extensive tissue fibrosis and scarring were postulated, while acute adverse events of primary RCT had already subsided [27].

Although various experts in the field currently consider this specific time window optimal, the definition of this interval is primarily based on clinical studies exploring overall survival and surgical complications after salvage surgery [27]. To the best of our knowledge, no direct assessment of alterations of the skeletal muscle composition in HNSCC patients after primary RCT has been performed yet.

Currently, various methods exist to explore RT- and chemotherapy-induced alterations of the skeletal muscle composition during and after RCT in HNSCC patients [28]. In addition to subjective methods, such as palpation, which are more of historical relevance, objective methods to assess the skeletal muscle composition in the neck were previously performed based on routinely acquired images during routine oncologic follow-up or small trials by using ultrasound or shear wave elastography [17,18,21,29]. Despite these attempts, available data applying these methods to assess RT-induced alteration of the skeletal muscle composition during and after RCT of HNSCC patients are sparse.

No attempt has yet been made to explore short-term RCT-induced alterations of the skeletal muscle composition after RCT in HNSCC patients applying a radiomic approach [16,17,18,21,28,29]. Radiomics is an emerging data-driven approach aiming for the extraction and processing of quantitative data to analyze image-based information [20]. The target is to treat the medical imaging data of patients as data-minable sources for additional clinical information. The result can provide an ameliorated basis for the decision-making process [19].

The primary aim of this study was to quantify tissue alterations in the skeletal muscles of the head and neck after primary RCT, applying radiomic feature analysis on routinely acquired CT images.

## 2. Materials and Methods

### 2.1. Study Population and Additional Clinical Data

This retrospective cohort study adhered to the “Strengthening the Reporting of Observation studies in Epidemiology” (STROBE) guidelines [30]. From 2008 until 2021, patients of the institutional head and neck cancer registry at the Department of Otorhinolaryngology, Head and Neck Surgery, Medical University of Innsbruck, Austria, (1) who had incident, histologically confirmed, locally advanced HNSCC (UICC III or IV), (2) who were treated with primary RCT, and (3) for whom contrast-enhanced computed tomography scans before treatment (“staging CT”) and after treatment (“restaging CT”) were available were eligible.

**Figure 1:**
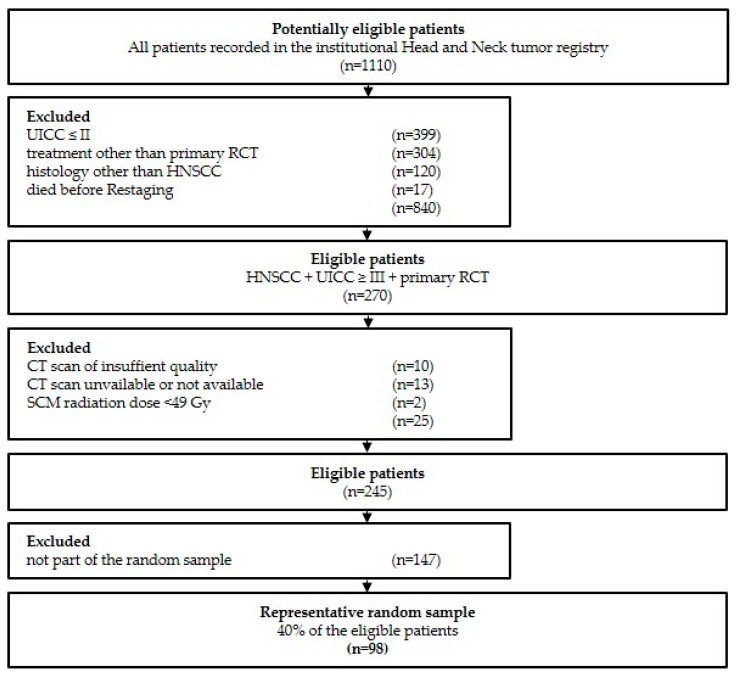
Study flow and patient inclusion modified according to STARD criteria [31]. A total of 1100 patients were potentially eligible, of which 840 did not meet the inclusion criteria. Of 270 eligible patients, 23 were excluded due to insufficient quality of the contrast-enhanced CT scan (e.g., dental artifacts) and 13 due to unavailability of either of the two imaging modalities. A representative random sample of 98 patients was drawn..

Of 1110 potentially eligible patients, 840 did not meet the inclusion criteria. From the remaining 270 patients, 25 were excluded due to the insufficient quality (e.g., dental artifacts) of the contrast-enhanced computed tomography scan (CT) prior to primary RCT (“staging CT”) and/or after primary RCT (“restaging CT) (n = 10), unavailability of either of the two imaging modalities (n = 13), or sternocleidomastoid muscles (SCM) radiation dose <49 Gy. Of the remaining 245 patients, a representative random sample was drawn with SPSS 28 (IBM, Armonk, NY, USA). The study flow diagram and patient inclusion modified according to the Standards for Reporting Diagnostic accuracy studies criteria (STARD) [31] are depicted in Figure 1.

### 2.2. Clinical Data

Clinical data were extracted from the institutional head and neck cancer registry or the electronic hospital information system (PowerChart, Cerner, Kansas City, MI, USA) including age, gender, year of first diagnosis, tumor site, UICC stage, date of the CT scans, alcohol consumption, smoking, radiation dose, p16 status, body mass index (BMI), serum protein level, American Association of Anesthesiologists (ASA) score, and functional outcome.

### 2.3. CT Imaging Acquisition

Staging and restaging contrast CTs adhered to the head and neck CT imaging protocols of the Department of Radiology (Medical University of Innsbruck, Austria) and were acquired with a Light Speed VCT or a Light Speed 16 CT scanner (GE Medical Systems, Vienna, Austria). The scan volume ranged from the skull base to the upper mediastinum with a resolution of 512 × 512 pixels, 2 mm slice thickness, collimation of 24 × 1.2 mm, and 0.45 pitch. Sagittal and coronal images were reconstructed from the axial images. As a contrast agent, Jopamiro 370 (Bracco Austria GmbH, Vienna, Austria) was administered intravenously, adjusted to the patient’s body weight. Staging and restaging contrast CTs used the same protocols, making the studies of each patient comparable.

### 2.4. Segmentation of Head and Neck Musculature

All staging and restaging CTs were first exported to the Digital Imaging and Communications in Medicine format using the IMPAX EE image archiving and communication system (Agfa HealthCare, Bonn, Germany). Thereafter, the images were exported and further processed using mint Lesion™ (Mint Medical GmbH, Heidelberg, Germany, version 3.8.1). For each patient, both the SCM and the paravertebral musculature (PVM), including the trapezius, longus capitis, splenius capitis, semispinalis capitis, longissimus capitis, levator scapulae, and longus colli, were segmented in staging and restaging CT.

The SCM, in the field of radiation and additionally affected by scatter irradiation, was chosen to explore RT-induced alterations, while the PVM, primarily affected by chemotherapy and less by scatter irradiation, was chosen to explore chemotherapy-induced alterations.

**Figure 2:**
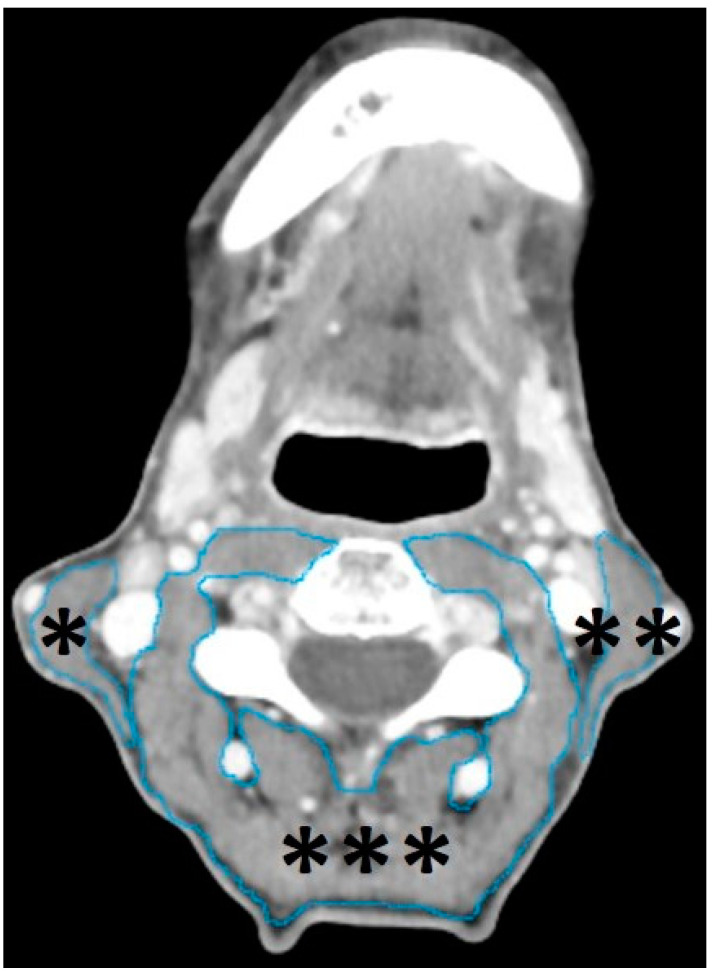
Example of segmented head and neck musculature in a staging CT of a 56-year-old male HNSCC patient with a tumor of the hypopharynx (not depicted), staged cT3 cN0 cM0. The manual slice-by-slice segmentation was performed in the axial plane using the “paint on slice” tool provided by mint Lesion™ (Mint Medical GmbH, Heidelberg, Germany, version 3.8.1) at the level of the third cervical vertebra, as previously proposed [32], for the right SCM (*), the left SCM (**), and the PVM (***), which included the following muscles: trapezius, longus capitis, splenius capitis, semispinalis capitis, longissimus capitis, levator scapulae, and longus colli.

The segmentations were performed manually in all data sets slice by slice in axial planes, using the “paint on slices” tool provided by the software, from the upper edge to the lower edge of the third cervical vertebra. This approach was previously found to be effective in the assessment of the head and neck musculature in HNSCC patients [32]. An example of so segmented head and neck musculature in a staging CT of a 56-year-old-male HNSCC patients with a tumor of the hypopharynx, staged cT3 cN0 cM0 is provided in Figure 2.

### 2.5. Data Analysis

At the time this study was conducted, mint Lesion™ provided 13 radiomic parameters for the three segmented muscle groups, SCM right, SCM left, and PVM, each before and after therapy. Normally distributed data were described by mean and standard deviation, and non-normally distributed parameters were described by median and 25th and 75th percentiles. Frequencies were tabulated and presented with percentages.

To reduce the number of radiomic parameters (initially 13), a principal component analysis (PCA) with Varimax rotation was performed. Here, three components could be extracted. Attempts with more components did not yield better results. The three extracted components were (1) volume, (2) mean pixel positivity (MMP), and (3) uniformity. Volume in milliliters (mL) represented the spatial dimension of the segmented muscles, SCM and PVM. A change in volume was considered as a surrogate for the loss of musculature (i.e., sarcopenia) or inflammation and interstitial edema. Mean positivity of pixels (MPP) in Hounsfield units (HU) represented an intensity parameter, which is a dimensionless absolute number. A change in MPP was considered as a surrogate for fibrosis (i.e., increase) or edema (i.e., decrease). Uniformity, a dimensionless absolute number, represented the texture of the segmented muscles, SCM and PVM. A change in uniformity was considered as a surrogate for changes in texture (i.e., fibrosis or edema).

These components had high loadings for the output values of volume, MPP, and uniformity provided by mint Lesion™ (Table 1; all > 0.9). Therefore, these three original values provided by mint Lesion™ were used for the present analyses. The remaining 10 radiomic parameters provided by mint Lesion™ correlated closely with one of these three radiomic features each. Thus, they were largely redundant and were not considered in further analysis.

These three key features represented the spatial dimensions, pixel intensity, and pixel uniformity of the segmented muscles.

The values of the three radiomic parameters, volume, MPP, and uniformity, were available for each of the three muscle groups (SCM left, SCM right, PVM) before and after therapy. Only the SCM data of the irradiated side were evaluated; if the radiation dose was the same on the right and left sides, the right SCM was used. The PVM was omitted from the radiation field and received the lowest possible radiation dose. The radiation dose to the irradiated SCM and the interval between diagnostic CT and restaging CT were recorded. In addition, data on BMI and serum protein level before and after therapy were available.

First, we tested for linear correlations (Pearson) between values before therapy for the radiomic parameters: volume, MPP, uniformity, patient age, BMI, and serum protein level. The influence of gender, tumor site, UICC stage, general health as measured by ASA score (I/II vs. III/IV), smoking status (≤ or > 10 pack years), and alcohol consumption (daily or less frequently than daily) on pretherapeutic parameters was examined by variance analysis.

The differences before and after therapy for the parameters of volume, MPP, uniformity, BMI, and serum protein level were tested with the paired-samples *t*-test (two-sided). Cohen’s d with Hedges’ correction was used as the effect size parameter. For further mechanistic analyses, the differences after therapy minus before therapy were calculated for these data and subjected to bivariate parametric correlation analyses and variance analysis. In addition, for the radiomic parameters, the results of the paired *t*-tests were adjusted for the effect of BMI difference by including BMI difference as a covariate.

### 2.6. Ethical Considerations

The study was approved by the review board of the Medical University of Innsbruck, Austria (1269/2018). All procedures conducted in these studies involving human participants were in accordance with the ethical standards of the institutional review board and with the Helsinki declaration (1964) and its later amendments or comparable ethical standards.

## 3. Results

### 3.1. Patient Population

A total of 1110 patients recorded in the institutional HNC registry were potentially eligible. Of these, 853 were excluded as they met one or more of the exclusion criteria (Figure 1). After drawing a representative random sample of the remaining 247 patients, 98 patients were included in this study.

Of these, 73 (74.5%) were male and 25 (25.5%) were female. Mean age at initial diagnosis was 62.0 (± 9.6) years, ranging from 42 to 81 years. Of the included 98 patients, the tumor site was oropharynx in 46 (46.9%), hypopharynx in 20 (20.4%), larynx in 15 (15.3%), oral cavity in 13 (13.3%), and another site in 4 (4.1%) patients. Of the 46 patients diagnosed with oropharyngeal HNSCC, 29 (63.0%) were categorized as p16 positive, using immunohistochemistry with a positivity cutoff of 60%..

In terms of comorbidities, 53 (54.1%) patients were classified as ASA III/IV and 45 (45.9%) as ASA I/II. All 98 (100.0%) included patients were smokers and 52 patients (53.1%) drank alcohol daily; the remaining 46 patients (46.9%) did not drink daily. BMI data were collected from 79 (80.6%) patients at initial diagnosis. The mean BMI was 24.2 (±4.9), ranging from 13.8 to 47.7. Thus, 7 (8.9%) patients were underweight, 41 were (51.9%) of normal weight, 25 (31.6%) were overweight, and 6 (7.6%) were defined as adipose. Additional details about clinical characteristics of the 98 included HNSCC patients are provide in the following table (Table 2).

### 3.2. Primary Radiochemotherapy and Time Intervals

All 98 included HNSCC patients were treated with primary RCT. All included patients received a radiation dose of at least 50 gray (Gy) on the irradiated and investigated SCM. For all included patients, the mean radiation dose for the SCM was 66.3 (±5.6) Gy, ranging from 50.0 to 70.8 Gy. For the present analysis, all included HNSCC patients were dichotomized into two groups, with radiation dose ≤60 Gy and >60 Gy for the SCM. For the 72 patients (73.5%) receiving a radiation dose of ≤60 Gy on the SCM, the mean radiation dose was 57.9 (±4.1), ranging from 50.0 to 60.0 Gy. For the 26 patients (26.5%) receiving a radiation dose of >60 Gy on the SCM, the mean radiation dose was 69.2 (±2.2), ranging from 60.1 to 70.8. Means, standard deviation, and range for the imaging interval (i.e., interval between staging CT and restaging CT) and restaging interval (i.e., interval between the end of primary RCT and restaging CT) are provided in Table 3.

### 3.3. Variable Reduction (Principal Component Analysis)

For dimensional reduction in the radiomic parameters yielded by mint Lesion™, a PCA with Varimax rotation was performed. From the 13 parameters of mint Lesion™, three principal components could be extracted. The three extracted components via PCA explained more than 70% of the variance of the values and were uncorrelated (Appendix A). The Kaiser–Meyer–Olkin Measure of Sampling Adequacy was 0.61, indicating an acceptable sampling adequacy.

The component matrix allowed the interpretation of the three extracted components based on their factor loadings (Appendix B). The first component represented a measure of the uniformity of the pixels in the segmented muscle, which was inversely correlated with entropy. The second component represented a measure of the pixel intensity, and the third component represented a measure of spatial dimension. Further detail about Variable Reduction via Principal Component Analysis is provided in Appendix C, Appendix D and Appendix E.

### 3.4. Factors Influencing Pretherapeutic Volume, Uniformity, and MPP

For comparisons, the right SCM was used as reference for the three segmented muscle groups. A correlation (Pearson) among muscle volume, BMI (r = 0.56; *p* < 0.001), and serum protein level (r = 0.31; *p* = 0.002) before therapy was observed. In addition, a weak inverse correlation between uniformity and age was observed (r = −0.21; *p* = 0.035).

Further comparisons (independent *t*-tests) revealed differences in gender. The pretherapeutic SCM volume on the right was 10.0 (±2.8) mL in 73 men and 5.9 (±1.7) mL in 25 women (*p* < 0.001). Differences according to gender were also observed for MPP, with 58.8 (±7.9) in men and 63.0 (±9.1) in women (*p* = 0.014), while there were no gender differences for uniformity. The other demographic and clinical factors (UICC stage, general health status (ASA score), smoking status (<10 PY/>10 PY), or alcohol consumption (daily or less frequently) did not affect the radiomic parameters explored.

### 3.5. Volume, Uniformity, and MPP before and after Therapy

The radiomic parameters for the SCM on the irradiated side of the neck that was exposed to the full radiation dose did not change except for volume (Table 4). Volume showed a decrease, from 9.00 (±3.2) to 8.4 (±2.7) mL (*p* = 0.007), with a Cohen’s d of 0.28, indicating a weak effect.

The SCM, in the field of radiation and additionally affected by scatter irradiation, was chosen to explore RT-induced alterations, while the PVM, primarily affected by chemotherapy and less by scatter irradiation, was chosen to explore chemotherapy-induced alterations.

The PVM, primarily affected by chemotherapy and less by scatter irradiation, was chosen to explore primarily for chemotherapy-associated muscular changes. However, changes analogous to those in the SCM were observed. The volume decreased from 96.5 (±30.2) to 91.9 (±25.8) (*p* = 0.007; Cohen’s d = 0.28). MPP and uniformity showed trends toward an increase but that may have been due to chance (*p* > 0.05, Table 4). Since the PVM was outside the radiation field, direct radiation exposure could not explain the observed volume decreases.

Consequently, it was tested whether the volume difference correlated with other parameters. Significant changes before and after therapy were seen in BMI, with a decrease from 23.9 (±4.2) to 20.98 (±3.59) kg/m^2^ (*p* ≤ 0.001; Cohen’s d = 0.9), and serum protein levels decreased from 7.4 (±0.53) to 6.75 (±0.85) mg/% (*p* < 0.001; Cohen’s d 0.7). Therefore, Pearson correlation analyses were included between volume differences before and after therapy and BMI difference, as well as the difference in serum protein levels before and after therapy. First, there was a significant correlation of the volume differences of the SCM and PVM (r = 0.58; *p* < 0.001). In addition, the volume decrease in the SCM and decrease in BMI correlated (r = 0.27; *p* = 0.007) as well as the volume decrease in the PVM musculature and BMI decrease (r = 0.41; *p* < 0.001). The decrease in serum protein levels did not correlate with the explored radiomic key parameters.

To test whether the radiomic volume decreases were due to the decrease in BMI, the results of the *t*-tests were adjusted for the difference in BMI values. For this purpose, general linear models completely analogous to the paired t-tests were used, with the BMI difference as a covariate. Here, the irradiated SCM showed a significant interaction between volume and BMI decrease (*p* = 0.007), whereas the difference before/after therapy was no longer significant (*p* = 0.9). Similarly, the PVM showed a significant interaction of volume decrease with BMI decrease (*p* < 0.001), and the volume decrease before/after therapy was no longer significant (*p* = 0.71).

## 4. Discussion

Multimodality treatment is frequently required in the treatment of locally advanced HNSCC [1,2,3,4]. Despite the precision of modern RT, which primarily targets the primary tumor and suspect cervical lymph nodes [5,6], undesired tissue alterations (i.e., fibrosis) especially in the skeletal muscle of the head and neck were previously observed [14,15,16,17,18]. These alterations occurred in the musculature that was directly in the field of radiation (i.e., SCM) but also in the musculature that was only indirectly affected by scatter irradiation (i.e., PVM) [14,15,16,17,18].

A link among the observed tissue alterations and the time interval between the end of RCT and the time of tissue assessment was previously postulated [14,15]. This postulated temporal linkage is of crucial importance in the context of salvage surgery, which is frequently necessary in the context of tumor or lymph node persistency after RCT [22,23,24,25,26]. The optimal time window to perform salvage surgery was previously clinically postulated by experts in the field at between 6 and 12 weeks after the completion of RCT [27]. For this specific time window, no extensive tissue fibrosis and scarring were found, while acute adverse events of primary RCT had already subsided [27].

Unfortunately, the definition of this specific time window for salvage surgery was mainly based on clinical studies exploring overall survival and surgical complications after salvage surgery [27]. Studies that directly assess tissue alterations in the skeletal muscle of the head and neck after RCT are sparse [17,18,21,29]. To the best of our knowledge, no attempt has yet been made to explore short-term RCT-induced alterations of the skeletal muscle after RCT in HNSCC patients applying radiomics, a data-driven approach aiming at the extraction and processing of quantitative data to analyze image-based information [19,20].

The primary aim of this study was to quantify tissue alterations in the skeletal muscles of the head and neck after primary RCT, applying radiomic feature analysis.

From a total of 247 eligible patients with locally advanced HNSCC recorded in the institutional HNC registry, a representative sample of 98 patients was drawn. Of these 98 patients, common clinical characteristics including sex, age, tumor site, p16 status, and smoking and drinking habits were comparable with previous, larger, cancer registry-based studies [33] (Table 2). Thus, the sample drawn from the original 247 eligible patients appears representative.

The imaging interval (i.e., the time between the pretreatment staging CT and the posttreatment restaging CT) was approximately 21 weeks, ranging from 15 weeks to 45 weeks (Table 3). In this specific interval diagnostic work-up, an interdisciplinary tumor board presentation and pretreatment procedures including dental treatments and the application of percutaneous gastrostomies were carried out. At our institution, the pretreatment procedures prior to the start of primary RCT require approximately 3 to 4 weeks, and primary RCT, an additional 6 to 8 weeks. Restaging was performed 6 to 12 weeks after the end of RCT. Thus, without complications during pretreatment procedures (e.g., percutaneous gastrostomy wound infections) or during primary RCT (e.g., postponing a radiochemotherapy cycle due to changes in white blood count), this interval ranges from 15 to 24 weeks. In the present study, the maximum interval observed was approximately 45 weeks. In this specific patient, a combination of pre- (peritonitis after percutaneous gastrostomy) and intratreatment complications (multiple postponements of radiochemotherapy cycles due to neutropenia) occurred.

The restaging interval (i.e., the time between the end of the RCT and the restaging CT) was approximately 8 weeks, ranging from 4 weeks to 33 weeks (Table 3). Thus, the mean time interval of the population explored in this study is in line with the recommended restaging interval, if additional salvage surgery is required [27]. The patient with the shortest restaging interval of 29 days was diagnosed with cT4a cN3b cM0 laryngeal cancer and therefore required urgent salvage laryngectomy. Thus, a considerably shorter restaging interval was observed for this patient. The patient with the longest restaging interval, of 229 days, was the one patient with multiple pre- and intratreatment complications, which required a prolonged intensive care unit stay after the completion of the primary RCT.

In summary, the intervals observed in the present study appear to be in line with intervals reported in previous studies [34,35,36]. Consequently, the observations of this study may be applied to the postulated optimal time window to perform salvage surgery by experts in the field at between 6 and 12 weeks after the completion of RCT [27].

The first key finding of the present study was that volume was the only radiomic key parameter that was subject to significant change after primary RCT. The mean SCM volume decreased from 9.0 to 8.4 mL and the PVM volume decreased from 96.5 to 91.9 mL (both *p* = 0.007). Some crucial aspects of this observation need to be discussed: considering the restaging interval of approximately 8 weeks (Table 3), an increase in volume due to muscular inflammation and interstitial edema in the SCM, directly affected by irradiation, was to be expected [16]. In addition, a significant decrease in mean PVM volumes was observed; it was primarily affected by chemotherapy but not by irradiation.

Consequently, it was considered unlikely that these observed changes in volume were primarily caused by irradiation, chemotherapy, or the combination of both treatment modalities alone. Moreover, additional, significant decreases in mean BMI from 23.9 to 21.0 kg/m² and in mean serum protein levels from 7.4 to 6.6 mg/% (both *p* < 0.001) were observed. These two parameters were included in Pearson correlation analyses, which revealed a significant and strong correlation for the BMI decrease (*p* < 0.001; r = 0.41). In a last step, the original results of the t-test applied to the volumetric changes were adjusted for the difference in BMI values, ultimately resulting in insignificant volume changes for the SCM and PVM after treatment (both *p* > 0.05). Other studies, such as that of Choi et al., came to a similar conclusion. However, in the aforementioned work, the method of measurement differed and contained significantly fewer parameters [37,38,39,40].

Thus, the second key finding of the present study was that no significant changes in the explored radiomic key features were observed, if adjusted for changes in BMI before and after treatment. This observation has several implications.

Firstly, this observation highlights the importance of an optimal assessment and the optimization of nutrition before, during, and after primary RCT. Regular assessments of nutritional status and BMI before, during, and after primary RCT for HNSCC patients were previously recommended by other authors [41]. In addition, both lower serum protein levels and lower BMI were previously linked to higher complication rates in HNSCC patients undergoing salvage surgery [42,43]. In a retrospective cohort study including 280 HNSCC patients, Danan and co-authors observed that lower albumin levels were associated with an increased rate of surgical complications and poorer overall survival [43]. In another multicenter retrospective review, including 33 institutions with 486 HNSCC patients, published by the Microvascular Committee of the American Academy of Otolaryngology-Head and Neck Surgery, a lower BMI was associated with a higher rate of surgical complications in patients undergoing salvage surgery [42]. These previously reported observations suggest that increased complication rates in patients undergoing salvage surgery later than the clinically postulated optimal time window of 6 to 12 weeks may additionally be affected by changes in BMI and serum protein levels and not solely by radiation-induced tissue alterations.

Secondly, the data of the present study support the previously clinically postulated optimal time window to perform salvage surgery [27]. The volume of the muscles explored did not significantly change, if corrected for changes in BMI, nor did the radiomic key features, MMP and uniformity, which can be considered as surrogates for tissue fibrosis.

Certain limitations of the present study need to be addressed. This comparatively small-numbered, retrospective study exploring only patients with advanced-stage HNSCC should be supplemented by a larger, prospective investigation. In addition, of the 245 potentially eligible patients, a representative sample of 98 patients (approximately 40%) was drawn. This decision was based on two aspects. Firstly, manual slice-by-slice segmentation with mint Lesion^TM^ is time consuming [34,36]. Secondly, according to the central limit theorem, normal distribution could be assumed with the current sample size. No significantly different outcomes were to be expected with a larger sample. In addition, limiting the sample size to the present 98 patients avoided *p*-value inflation. Various data processing programs are available to segment anatomical structures. For the present study, the commercially available software mint Lesion™ was chosen due to the advantage of providing a structured, standardized feature output available to everyone, which minimizes the risk of bias. However, at the time of the study, only 13 radiomic features were routinely extracted by mint Lesion^TM^. Furthermore, these 13 features were reduced to three key features via PCA. Despite applying machine learning or deep neural networks for statistical analysis, attempts with more components did not yield better results [44]. Thirdly, the time interval chosen for the present study only explored for short-term radiomic changes with a mean restaging interval of approximately 8 weeks. An expansion of this observation interval to months or years would be crucial to assess whether previously proposed changes of the head and neck musculature due to primary RCT can be detected by means of a radiomic approach.

## 5. Conclusions

After a mean interval of approximately 8 weeks after the completion of primary RCT, no significant radiomic changes could be assessed. The data of the present study support the previously clinically postulated optimal time window to perform salvage surgery of 6 to 12 weeks.

## Figures and Tables

**Table 1:** All 13 individual radiomic features and their corresponding radiomic key feature extracted with mint Lesion^TM^ (Mint Medical GmbH, Heidelberg, Germany, version 3.8.1).

Radiomic Key Features	Individual Radiomic Features
Shape features	Short axis diameter ^1^ Long axis diameter ^1^ Volume ^2^
Texture features ^3^	Entropy Uniformity
Intensity features ^3^	Maximal density Minimal density Mean density Skewness of density Standard deviation of density MPP Uniformity of distribution of positive pixels (UPP) Kurtosis

^1^ Diameters are provided in millimeters; ^2^ volumes are provided in milliliters; ^3^ all texture and intensity features are provided without dimension.

**Table 2:** Clinical characteristics of the 98 included HNSCC patients.

		Number	Percentages
**Sex**	Male	73	74.5%
	Female	25	25.5%
**Age**	≤50	11	11.2%
	51–60	35	35.7%
	61–70	33	33.7%
	≥71	19	19.4%
**Tumor site**	Oral cavity	13	13.3%
	Oropharynx	46	46.9%
	Hypopharynx	20	20.4%
	Larynx	15	15.3%
	CUP ^1^	4	4.1%
**UICC ^1^ truncated**	Stage III	19	19.4%
	Stage IV	79	80.6%
**ASA**	ASA I/II	45	45.9%
	ASA III/IV	53	54.1%
**Alcohol consumption**	<Daily	52	53.1%
	Daily	46	46.9%
**Smoking habits**	<10 PY	24	24.5%
	≥10 PY	74	75.5%
**BMI classified**	Underweight	7	8.9%
	Normal weight	41	51.9%
	Overweight	25	31.6%
	Adipose	6	7.6%
**Radiation dose ^2^**	≤60 Gy	26	26.5%
	>60 Gy	72	73.5%

^1^ Carcinoma of unknown primary; ^2^ dosage in Gy on the investigated SCM.

**Table 3:** Time intervals of the 98 included HNSCC patients between staging CT and restaging CT (imaging interval) as well as between end of RCT and restaging CT (restaging interval).

	Mean (Days)	Minimum (Days)	Maximum (Days)	Standard Deviation (Days)
**Imaging interval ^1^**	148	108	315	±33
**Restaging interval ^2^**	55	29	229	±28

^1^ Imaging interval: interval between staging CT and restaging CT in days; ^2^ restaging interval: interval between end of RCT and restaging CT in days.

**Table 4:** Radiomic key parameters extracted via mint Lesion^TM^ in staging CTs (i.e., pretreatment) and approximately 21 weeks later in restaging CTs (i.e., posttreatment) in the included 98 patients with incident, locally advanced HNSCC.

Radiomic Key Features	Staging CT (SD)	Restaging CT (SD)	*p*-Value ^1^	Cohen’s d ^2^
SCM Volume (mL)	9.00 (±3.2)	8.4 (±2.7)	0.007	0.28
SCM MPP (HU)	60.1 (±8.7)	59.7 (±8.1)	0.664	0.04
SCM Uniformity *	16.8 (±4.3)	16.4 (±4.1)	0.342	0.10
PVM Volume (mL)	96.5 (±30.2)	91.9 (±25.8)	0.007	0.28
PVM MPP (HU)	56.3 (±8.6)	58.0 (±8.6)	0.061	−0.19
PVM Uniformity *	11.6 (±3.1)	12.0 (±2.8)	0.058	−0.19

^1^ Two-sided paired-sample *t*-test; ^2^ Cohen’s d with Hedges’ correction; * values provided multiplied by 1000; SD: standard deviation.

## Data Availability

The data presented in this study are available on request from the corresponding author.

## Appendix A

**Table A1:** Descriptive statistics of the original obtained values by mint Lesion^TM^ for the SCM and PVM. In addition, the data for the SCM of the radiated side (SCM-rad) are provided. These data were obtained before treatment.

	N	Minimum	Maximum	Mean	Standard Deviation
SCMright_Short_Axis	98	4.7	24.3	14.251	3.4045
SCMright_Long_Axis	98	19.5	60.9	42.026	7.5338
SCMright_Volume	98	3.3	17.8	8.971	3.1417
SCMright_Entropy	98	5.5	7.3	6.489	0.3232
SCMright_Kurtosis	98	3.1	24.4	6.557	2.7009
SCMright_MPP	98	31.6	79.2	59.880	8.3491
SCMright_Density_Max.	98	72	234	137.21	36.768
SCMright_Density_Min.	98	−155	−66	−105.32	17.386
SCMright_Density_Mean	98	23.8	77.5	51.176	10.9033
SCMright_Density_Skewness	98	−3.0	−1.0	−1.75	0.373
SCMright_Density_SD	98	13.9	45.3	32.183	5.9952
SCMright_UPP	98	0.0076	0.0298	0.016484	0.0041614
SCMright_Uniformity	98	0.0078	0.0298	0.016621	0.0040749
SCMleft_Short_Axis	98	5.0	27.7	14.547	3.7930
SCMleft_Long_Axis	98	23.8	57.5	42.623	7.6126
SCMleft_Volume	98	2.9	15.7	8.930	3.0336
SCMleft_Entropy	98	5.5	7.3	6.494	0.3395
SCMleft_Kurtosis	98	2.0	14.6	6.262	2.0012
SCMleft_MPP	98	37.1	86.0	59.344	8.4566
SCMleft_Density M_Axis	98	73	200	138.57	28.363
SCMleft_Density_Min.	98	−178	−48	−105.65	18.427
SCMleft_Density_Mean	98	23.6	78.6	50.546	10.9604
SCMleft_Density_Skewness	98	−3.4	−0.8	−1.717	0.4310
SCMleft_Density SD	98	14.1	47.9	32.052	6.5302
SCMleft_UPP	98	0.0075	0.0291	0.016344	0.0043101
SCMleft_Uniformity	98	0.0078	0.0291	0.016514	0.0042213
PVM_Short_Axig	98	43.7	88.9	66.209	10.0206
PVM_Long _Axis	98	63.3	176.3	100.281	18.8547
PVM_Volume	98	34.4	183.9	96.452	30.1608
PVM_Entropy	98	5.9675	7.5142	6.959589	0.2929190
PVM_Kurtosis	98	1.20	32.40	7.1407	4.72660
PVM_MPP	98	34.9	77.2	56.324	8.6289
PVM_Density_Max.	98	220.0	1278.0	520.678	211.4958
PVM_Density Min.	98	−198	−86	−127.35	19.423
PVM_Density_Mean	98	11.9	70.0	43.765	12.7727
PVM_Density_Skewness	98	−2.1	3.0	−0.735	0.8544
PVM_Density_SD	98	20.1	61.8	39.053	7.0062
PVM_UPP	98	0.0057	0.0235	0.011340	0.0032782
PVM_Uniformity	98	0.0067	0.0235	0.011584	0.0030456
SCM-rad_Volume	98	3.30	17.80	8.9992	3.15632
SCM-rad_Entropyopy	98	5.45	7.28	6.4731	0.33946
SCM-rad_MPP	98	31.60	86.00	60.1269	8.70200
SCM-rad_Density Mean	98	23.60	78.60	51.9051	11.08669
SCM-rad_Uniformity	98	0.01	0.03	0.0168	0.00425

## Appendix B

**Table A2:** Matrix of observed Pearson correlation coefficients (here: right SCM); n = 98; bold numbers indicate correlation coefficients between ±0.5 and ±0.99.

	Short _Axis	Long _Axis	Volume	Entropy	Kurtosis	MPP	Density_Max.	Density_Min.	Density_Mean	Density_Skewness	Density_SD	UPP	Uniformity
Short_Axis	1.00	0.11	0.45	−0.12	0.12	0.06	0.00	0.04	0.13	−0.12	−0.16	0.10	0.09
Long_Axis	0.11	1.00	**0.72**	−0.09	0.12	−0.03	0.04	−0.04	0.01	−0.13	−0.07	0.10	0.10
Volume	0.45	**0.72**	1.00	−0.12	0.13	−0.06	−0.03	−0.07	−0.01	−0.16	−0.12	0.12	0.11
Entropy	−0.12	−0.09	−0.12	1.00	**−0.72**	−0.04	0.28	**−0.58**	−0.37	**0.70**	**0.87**	**−0.96**	**−0.96**
Kurtosis	0.12	0.12	0.13	**−0.72**	1.00	0.27	0.02	0.18	0.46	**−0.76**	**−0.57**	**0.75**	**0.75**
MPP	0.06	−0.03	−0.06	−0.04	0.27	1.00	0.35	0.22	**0.90**	−0.11	0.06	0.12	0.11
Density M_Axis	0.00	0.04	−0.03	0.28	0.02	0.35	1.00	−0.13	0.27	0.24	0.18	−0.25	−0.25
Density Min	0.04	−0.04	−0.07	**−0.58**	0.18	0.22	−0.13	1.00	0.47	−0.01	**−0.68**	0.48	0.47
Density Mean	0.13	0.01	−0.01	−0.37	0.46	**0.90**	0.27	0.47	1.00	−0.25	−0.35	0.38	0.36
Density Skew	−0.12	−0.13	−0.16	**0.70**	**−0.76**	−0.11	0.24	−0.01	−0.25	1.00	0.40	**−0.76**	**−0.76**
Density STD	−0.16	−0.07	−0.12	**0.87**	**−0.57**	0.06	0.18	**−0.68**	−0.35	0.40	1.00	**−0.73**	**−0.72**
UPP	0.10	0.10	0.12	**−0.96**	**0.75**	0.12	−0.25	0.48	0.38	**−0.76**	**−0.73**	1.00	1.00
Uniformity	0.09	0.10	0.11	**−0.96**	**0.75**	0.11	−0.25	0.47	0.36	**−0.76**	**−0.72**	1.00	1.00

## Appendix C

**Table A3:** Data variance explained by the three extracted factors.

Component	Sums of Squared Loadings	% of Variance	Cumulative %
Uniformity	5.3	40.8	40.8
Intensity	2.2	16.6	57.4
Dimension	2.0	15.2	72.6

## Appendix D

**Table A4:** Matrix of the three extracted components and the rotated factor loadings of the initial parameters. Principal component analysis, Varimax rotation with Kaiser normalization.

Factor Loadings	Uniformity	Intensity	Dimension
Entropy	−0.99		
UPP	0.97		
Uniformity	0.97		
Density SD	−0.84		
Kurtosis	0.77		
Density Skewness	−0.74		
Density Min.	0.57		
MPP		0.94	
Density Mean	0.41	0.88	
Density Max.	−0.31	0.62	
Volume			0.93
Long_Axis			0.82
Short_Axis			0.54

## Appendix E

**Table A5:** Pearson correlation coefficients (r) and corresponding two-sided probabilities (*p*) between the three components (factors) obtained by principal component analysis and the original parameters obtained by mint Lesion^TM^ with high factor loadings. For simplicity, these three original parameters were used instead of the three components.

		Dimension-Factor	Uniformity-Factor	Intensity-Factor	Original Volume	Original Uniformity	Original Intensity (MPP)
Dimension-Factor	r	1	0.08	0.017	0.937	0.165	0.018
	*p*		0.435	0.865	0.001	0.104	0.858
Uniformity-Factor	r		1	0.268	0.075	0.966	0.317
	*p*			0.008	0.462	0.001	0.001
Intensity-Factor	r			1	−0.074	0.228	0.963
	*p*				0.468	0.024	0.001
Original Volume	r				1	0.155	−0.057
	*p*					0.127	0.575
Original Uniformity	r					1	0.284
	*p*						0.005
Original Intensity	r						1
(MPP)	*p*						
